# Detection of Diuretics Contamination in Whey Protein-Based Dietary Supplements

**DOI:** 10.1021/acsomega.5c09357

**Published:** 2026-02-05

**Authors:** Inélia Maria Franskoviaki, Pâmela Cristina Lukasewicz Ferreira, Vanessa Klimkowski Argoud, Pedro Eduardo Froelich, Aline Rigon Zimmer

**Affiliations:** Graduate Program of Pharmaceutical Science, Faculty of Pharmacy, 28124 Universidade Federal do Rio Grande do Sul (UFRGS), 2752 Ipiranga Avenue, 90610-000 Porto Alegre, RS, Brazil

## Abstract

Whey protein dietary supplements (WPDS) are widely used by athletes at all levels to improve recovery between training sessions and competitions. The increasing number of reported cases of athletes who consumed these products and subsequently tested positive in antidoping tests has raised concerns that such products may contain undeclared substances. Diuretics are among the most common adulterants found in dietary supplements, and the presence of any amount of these substances in urine or blood samples is classified as doping. This study aimed to develop a reliable method using high-performance liquid chromatography with UV detection (HPLC-UV) to simultaneously detect the presence of the diuretics chlorothiazide, hydrochlorothiazide, furosemide, amiloride, and chlorthalidone in WPDS. A simple, selective, precise, and accurate method was validated according to ANVISA, FDA, and forensic toxicology guidelines using a C8 column, a 100 mM ammonium acetate buffer, and an acetonitrile mobile phase in a gradient system, with UV detection at 276 nm. The method was applied to the analysis of 21 commercial WPDS samples, and 42.9% tested positive for at least one of the investigated diuretics. Furosemide was the most frequently detected diuretic found in 28.8% (0.20–1.82 mg/g of WPDS) of all analyzed samples, followed by hydrochlorothiazide, detected in 19.0% (0.25–1.40 mg/g of WPDS). These results highlight the importance of screening for adulterants in WPDS before consumption to ensure proper quality control, protect consumer safety, and prevent unintentional doping cases.

## Graphical abstract

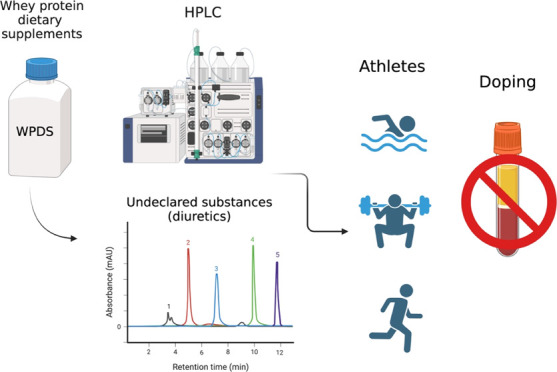

## 1 Introduction

Whey protein-based dietary supplements (WPDS) are extracted from milk whey during cheese production. These supplements have high levels of essential amino acids (30–36 g/100 g) and protein (70–80 g/100 g) are widely used by both professional and amateur athletes as performance-enhancing products.^1,2^ Since dietary supplements are meant to complement the diet of healthy individuals, these products are exempt from registration in Brazil by the National Health Surveillance Agency (ANVISA), as long as they do not contain enzymes or probiotics in their composition.^3^ However, they must comply with current legislation regarding their chemical composition and mode of use. Additionally, the label of these products must include the nutritional information and a list of all ingredients. WPDS also cannot contain substances with medicinal actions, such as hormones, stimulants, or any other substances that could be considered doping, including diuretics.^4^

The goal of using protein supplementation is to maximize muscle response and support skeletal muscle repair after exercise.^5^ WPDS is consumed by various categories of professional and amateur athletes. Although there is no well-defined daily dosage in the literature,^2^ manufacturers recommend a daily intake of 20 to 40 g. However, professional athletes should be aware that some dietary supplements may cause adverse effects or contain substances classified as doping agents, which are not declared on the label.^6–8^

The International Olympic Committee (IOC), along with the World Anti-Doping Agency (WADA), defines doping as any prohibited substance or its metabolites or markers found in athletes’ samples that are used to enhance performance.^9^ The Brazilian Doping Code considers doping as the presence of banned substances in athletes’ biological samples and outlines penalties.^10^

Diuretic agents are listed in the prohibited list by the WADA, and are not allowed in sports in- and out-of-competition because of their ability to increase diuresis and decrease body fluids, leading to temporary and artificial weight loss, as well as their possible masking effects on other doping agents in urine samples.^11,12^ The use of these substances for rapid, acute weight loss occurs mainly in sports stratified by weight categories, such as judo, karate, boxing, bodybuilding, and Olympic gymnastics, among others.^13^ The use of diuretics to dilute urine samples for antidoping tests can occur in any sport modality.^13,14^ This practice aims to make the detection of banned substances more difficult, either by dilution or by altering urinary pH and inhibiting renal passive reabsorption.^11,15^ Consequently, increased diuresis and urine dilution can dramatically decrease the concentration of illicit substances in urine, potentially leading to false-negative results.^11,14^

Cases of doping involving diuretics and masking agents have increased over time, including the unintentional doping resulting from the intake of dietary supplements containing unlisted banned substances. There are several documented cases in the scientific literature of dietary supplements (involving whey-protein-based products) contaminated or adulterated with undeclared substances, including prohibited diuretics.^16–21^ Currently, this category of drugs represents the second most common group of substances detected in antidoping tests conducted by WADA, surpassed only by anabolic steroids. Within the class of diuretics and masking agents, the most frequently detected substance is furosemide.^9,22^ However, a recent analysis conducted by the Brazilian Doping Control Laboratory, between 2017 and 2022, on dietary supplements revealed an alarming number of tainted samples, with diuretics among the most common adulterants across all supplement types.^23^

The presence of diuretics in any amount in urine or blood samples is considered doping and may result in antidoping violations and penalties for the athlete (WADA). Such contamination may occur intentionally or result from manufacturing lines with cross-contamination and inadequate quality control, since the regulation of dietary supplements generally does not require mandatory analytical testing in most countries.^17,20,21^ In the literature, analytical methods have been described for the simultaneous determination of several diuretics in dietary supplements and in complex matrices such as capsules, plasma, and urine.^17–19,24–28^ Most analyses, however, are performed in urine and plasma samples, with liquid chromatography coupled to mass spectrometry (LC-MS) being the main analytical method, despite its high operational cost.^17,19,24^ To date, limited analytical methods for the simultaneous determination of different classes of diuretics in WPDS using high-performance liquid chromatography with ultraviolet detection (HPLC-UV) have been reported.

In this context, considering the consequences of unintentional doping cases linked to dietary supplement use, this study aims to develop a simple and reliable HPLC-UV analytical method to identify and quantify diuretics in WPDS products, providing an accurate and precise technique for routine laboratory quality control that complies with regulatory requirements.

## 2 Materials and Methods

### 2.1 Reference Chemicals and Reagents

Reference standards of the diuretics amiloride (AML), hydrochlorothiazide (HCTZ), chlorothiazide (CTZ), chlorthalidone (CTD), and furosemide (FRS), all with purity >99.0%, were purchased from Sigma-Aldrich (St. Louis, MO, USA) and used for validation of the analytical method. Acetonitrile (ACN), methanol (MeOH), acetic acid (CH_3_COOH), and ammonium acetate buffer of HPLC grade were purchased from Merck (Frankfurt, Germany). Ultrapure water was obtained using a Milli-Q Plus system (Millipore, Bedford, MA, USA).

### 2.2 Sample Preparation

#### 2.2.1 Standard Solutions

The reference stock solutions of the diuretics AML, CTZ, HCTZ, FRS, and CTD were prepared in MeOH at a concentration of 2 mg/mL. Bromazepam was used as an internal standard (IS) at a final concentration of 100 μg/mL. All solutions were filtered through a 0.45 μm membrane filter (PVDF membrane Millex-GV Millipore) and stored in amber flasks under refrigeration.

#### 2.2.2 Pool of Drug-Free Matrix

The drug-free matrix used for the development and validation of the analytical method consisted of a mixture of WPDS samples free of the target diuretics, previously analyzed at the Secondary Standards Production Laboratory (LAPPS), Federal University of Rio Grande do Sul (UFRGS).

#### 2.2.3 Diuretics Extraction from the Matrix

A 500 mg portion of the drug-free matrix was weighed and spiked with six different concentrations of the reference stock solution containing the diuretics AML, CTZ, HCTZ, FRS, and CTD (20, 60, 100, 140, 180, 220 μg/mL) along with a fixed concentration of IS (100 μg/mL). Subsequently, 5.0 mL of MeOH was added to the mixture, stirred for 2 min, and then placed in an ultrasonic bath for 30 min. The mixture was centrifuged at 2500 rpm for 15 min, and the supernatant was collected and filtered through a 0.45 μm Millipore syringe filter (Merck) prior to injection into the HPLC-UV system.

### 2.3 High Performance Liquid Chromatography System

Analyses were performed on a SHIMADZU LC-20AT chromatograph equipped with a UV-DAD detector (model SPD-M1010 AVvp), degasser DGU-20A3, and autosampler, coupled to a SHIMADZU oven (model CTO-20A). Chromatographic separation was carried out using a C8 column (4.6 × 250 mm, 5 μm particle size; Phenomenex, USA) with a C8 precolumn. The injection volume was 20 μL, detection was performed at 276 nm, the flow rate was 0.9 mL/min, and the column temperature was maintained at 35 °C. The mobile phase consisted of ACN/100 mM ammonium acetate buffer, pH 4.5; the gradient conditions are shown in Table 1. Data analysis was performed using LC-Solutions software.

**Table 1:** Elution Conditions of the Mobile Phase under Gradient Mode in the HPLC-UV System

elution time (min)	ammonium acetate buffer pH 4.5 (%)	acetonitrile (%)
0.01	90	10
4.0	87	13
15	65	35
20	60	40
23	65	35
26	87	13
30	90	10
35	90	10

### 2.4 Analytical Method Validation

The validation of the analytical method was conducted in accordance with the guidelines established by ANVISA,^29^ further complemented by the Food and Drug Administration (FDA) protocol,^30^ and the standard practices for method validation in forensic toxicology.^31^ The HPLC-UV analytical methodology was validated according to the parameters of selectivity, specificity, linearity, limit of detection (LOD), limit of quantification (LOQ), precision, accuracy, robustness, and matrix effect. The diuretics of interest were extracted from the supplemented matrix as described in Section 2.2.3.

The method’s selectivity was assessed by comparing the retention time (*R*_*t*_) of each diuretic extracted from the matrix with the *R*_*t*_ obtained by injecting standard solutions containing the diuretics individually or in combination with all other diuretics of interest and the internal standard (IS) into the HPLC system. Additionally, the method’s specificity was evaluated by examining the UV spectral profile of each diuretic and assessing peak purity using LC-Solutions software.

The internal standard (IS) method was used for diuretic quantification. Calibration curves were prepared by spiking the matrix with six different concentrations of the diuretics of interest (20, 60, 100, 140, 180, 220 μg/mL) along with a fixed concentration of IS (100 μg/mL). To evaluate linearity, the compounds of interest were extracted from the matrix, and replicates of each calibration level were analyzed on three different days. The results were plotted as the diuretic concentrations in μg/mL (*X*-axis) versus the ratio of the mean areas of the diuretics to the IS (*Y*-axis). The Pearson correlation coefficient (*r*) was determined through linear regression analysis using the least-squares method and validated by analysis of variance (ANOVA).

The limit of detection (LOD) and limit of quantification (LOQ) were estimated using eqs 1 and 2, respectively. The data for these calculations were obtained from calibration curves analyzed on different days and were determined individually for each diuretic. LOQ was also confirmed experimentally for each diuretic by injecting the estimated concentration in triplicate using the validated methodology.

1 $$LOD=\left({SD}_{y-intercept}\times 3.3\right)/IC$$

2 $$LOQ=\left({SD}_{y-intercept}\times 10\right)/IC$$

SD: average standard deviation of the *y*-intercept of three calibration curves.

IC: inclination of the average curve of the three calibration curves.

The method’s precision and recovery were evaluated at three concentration levels: low (60 μg/mL), medium (120 μg/mL), and high (220 μg/mL), with five replicates at each concentration. Analyses were conducted on three different days, resulting in a total of 45 extracted solutions. The coefficient of variation (CV %) was calculated to assess intraday repeatability and interday precision. Results were compared using analysis of variance (ANOVA, α = 0.05). For acceptance, the mean CV % value was required to be below 15%.

The recovery was calculated according to eq 3.

3 $$C_{rec}=\left({{RA}_{ext}}^{*}C_{st}\right)/{RA}_{st}$$

*C*_rec_ = recovered concentration of each diuretic from the matrix.

RA_rec_ = ratio between the peak area of the diuretic of interest and the peak area of the IS extracted from the matrix.

*C*_st_ = concentration of the standard solution of the diuretic of interest.

RA_st_ = ratio between the peak area of the diuretic of interest and the peak area of the IS in the standard solution.

The robustness of the analytical method was evaluated by making slight changes to the eluent flow rate (0.8 and 1.0 mL/min) and oven temperature (33 and 37 °C). Each chromatographic condition was analyzed in triplicate using a standard solution at a concentration of 100 μg/mL.

The mean of three calibration curves prepared in the matrix supplemented with each diuretic was compared with the mean of three calibration curves prepared using the standard solution of each diuretic (20, 60, 100, 140, 180, 220 μg/mL, and IS) to assess the matrix effect. Parallelism of the curves was evaluated using a *t*-test on the variance of the regression lines’ slopes, with a significance level of 95%.^32^

### 2.5 Stability of Extracted Samples

The stability of the analytes after extraction from the matrix was assessed under three conditions: on the benchtop (25 °C) for 48 h, in the autosampler (35 °C) for 30 h, and in the refrigerator (4–8 °C) for 7 days. Processed samples at low (60 μg/mL), medium (120 μg/mL), and high (220 μg/mL) concentrations were analyzed in triplicate. After each storage period, samples were injected into the HPLC-UV system, and the recovery of each diuretic was calculated and compared with that of samples injected immediately after preparation.

### 2.6 Analysis of the Commercial Samples

To evaluate the applicability of the developed analytical method, 21 commercial WPDS samples were collected from dietary supplement users in Porto Alegre/RS, Brazil. Five hundred milligrams of each sample were homogenized in a mortar, and 0.5 mL of the IS stock solution was added to achieve a final concentration of 100 μg/mL. The mixture was then extracted as described in Section 2.2.3. A standard solution containing the diuretics under study and the IS was injected simultaneously. The concentration of diuretics in the positive commercial samples was determined according to eq 3.

## 3 Results and Discussion

### 3.1 Analytical Method Development and Validation

The HPLC-UV method is widely used in the industry for quality control and is recommended by official guidelines.^31,33^ In this study, an HPLC-UV method was developed and validated to determine the diuretics AML, CTZ, HCTZ, CTD, and FRS as potential contaminants in WPDS.

A single analysis wavelength of 276 nm was selected to develop a simple and accessible method for routine quality control, eliminating the need for a photodiode array (DAD) or mass spectrometry detector, as all diuretics exhibited adequate absorption at this wavelength (Figure S1). Additionally, this wavelength minimized interference from matrix components (Figure 1). A gradient system of ACN and 100 mM ammonium acetate buffer (pH 4.5) was employed to achieve improved peak resolution among the analytes of interest.

**Figure 1:**
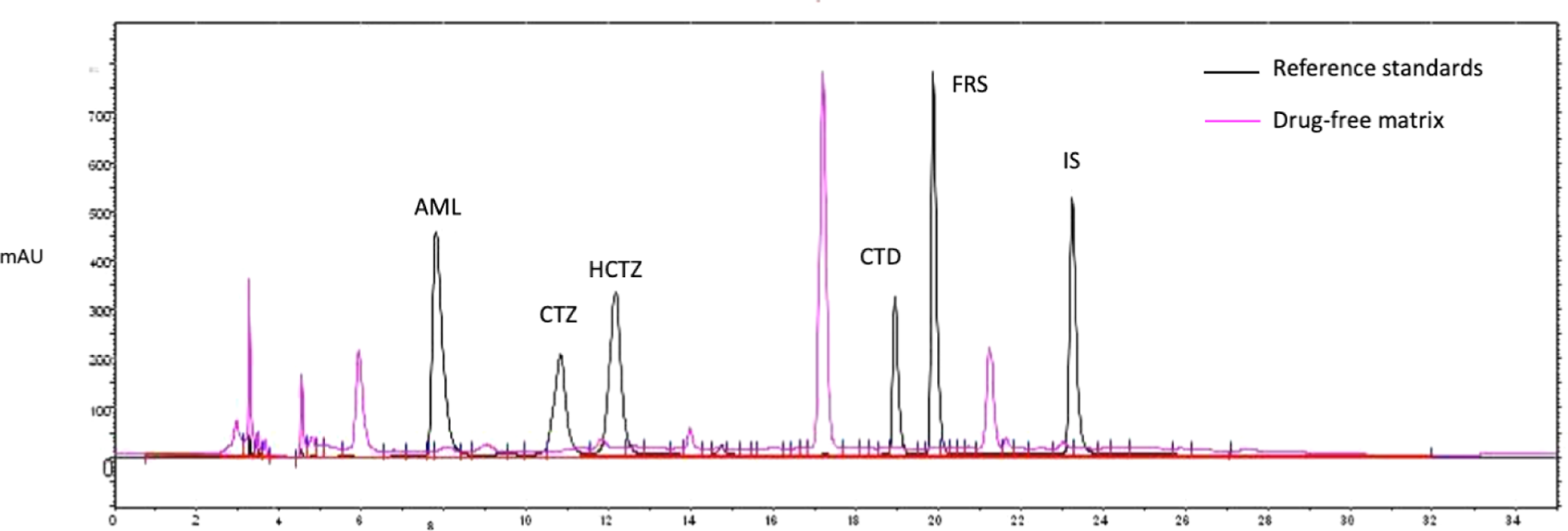
Chromatographic profile of the drug-free matrix (pink line) and the diuretics reference standards (black line) showing no overlap of the matrix peaks and the diuretics analyzed (200 μg/mL): AML = Amiloride, retention time (*R*_*t*_) = 7.90 min; CTZ = Chlorothiazide, *R*_*t*_ = 11.21 min; HCTZ = Hydrochlorothiazide, *R*_*t*_ = 12.46 min; CTD = Chlorthalidone, *R*_*t*_ = 19.03 min; FRS = Furosemide, *R*_*t*_ = 19.94 min; IS = Bromazepam, *R*_*t*_ = 23.27 min.

Selecting a sample preparation procedure for a complex matrix is always challenging due to the presence of interfering substances. Solid–liquid extraction followed by centrifugation proved to be a practical strategy for routine quality control, as it is fast and easy to perform. Methanol (MeOH) was chosen as the extraction solvent because it is selective for the diuretics of interest and is widely used for extracting adulterants from dietary supplements. Samples were therefore extracted with MeOH, vortexed for 2 min, subjected to an ultrasonic bath for 30 min, and centrifuged at 2500 rpm for 15 min, yielding a clear supernatant ready for injection after filtration.

Each diuretic was identified by comparing the retention time (*R*_*t*_) of individual reference standard solutions and by peak purity analysis, as shown in Table 2. Peak purity was determined by a DAD detector in the HPLC system through the comparison of UV spectra at several points across the chromatographic peak and calculation of the correlation coefficient (*r* ^2^) between them. Since all spectra were highly correlated (*r* > 0.99), the peak was considered pure, indicating that the chromatographic peak corresponds to a single analyte.

**Table 2:** Retention Time (*R*_*t*_) and Peak Purity of Substances of Interest in the Study

substance of analysis	*R*_*t*_ (min)	purity peak
amiloride	7.54	0.991
chlortiazide	11.21	0.997
hydrochlortiazide	12.46	0.998
chlorthalidone	19.00	0.998
furosemide	19.94	0.999
IS^a^	23.27	0.999

a Bromazepam: internal standard (IS).

Figure 1 presents the chromatographic profile of the reference standard diuretic mixture, showing adequate separation of each analyte peak (black line), alongside the peaks from the extracted drug-free matrix (pink line), injected separately. Figure 2A shows the chromatographic profile of the extraction sample from a drug-free matrix supplemented with reference standards of the diuretics (AML, CTZ, HCTZ, CTD, FRS, and IS). The method demonstrated selectivity, with no matrix interference observed in the peak purity of the diuretics of interest.

**Figure 2:**
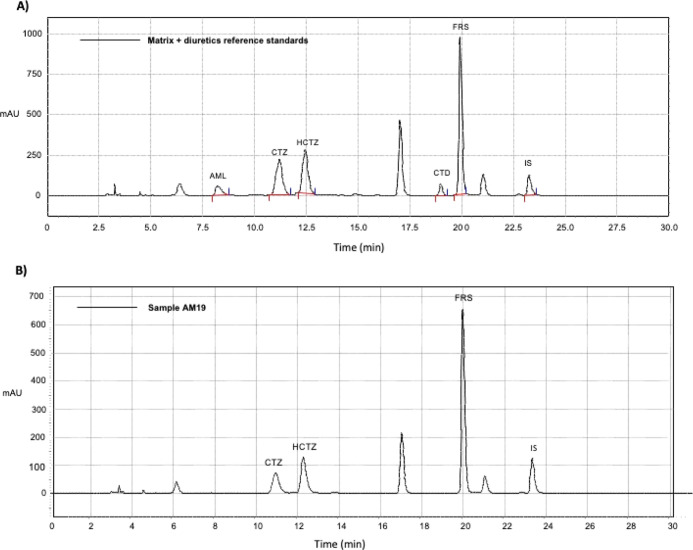
(A) Chromatographic profile of the extraction product from drug-free matrix supplemented with reference standards of the diuretics amiloride (AML), chlorothiazide (CTZ), hydrochlorothiazide (HCTZ), chlorthalidone (CTD), furosemide (FRS), and bromazepam (IS) showing the peak resolution of the analyzed compounds. Concentration of 150 μg/mL for the analytes and 100 μg/mL for the IS. (B) Chromatogram of a commercial whey protein dietary supplement sample (AM19) with positive result for three diuretics: chlorothiazide (CTZ), hydrochlorothiazide (HCTZ) and furosemide (FRS).

According to official guidelines, the LOD represents the lowest amount of an analyte that can be detected in a sample, whereas the LOQ corresponds to the lowest amount that can be quantified under experimental conditions.^30,32^ The method proved to be sensitive for detecting diuretics, with an LOD below 3.47 μg/mL. The methodology was most sensitive for CTD, which exhibited the lowest LOD and LOQ (1.39 and 4.21 μg/mL, respectively), and least sensitive for FRS, which showed the highest LOD and LOQ values (3.47 and 10.51 μg/mL, respectively) (Table 3). The LOQ values were confirmed experimentally, demonstrating suitable precision and accuracy for diuretic quantification (Figure S2).

**Table 3:** Linear Regression Curve, Correlation Coefficient (*r*), Standard Deviation (SD) of the *y*-Intercept, Slope of the Linear Regression, Limit of Detection (LOD), and Limit of Quantification (LOQ) Obtained for Each Analyte of Interest after Method Validation

substance of analysis	regression line	correlation coefficient (*r*)	SD of the *y*-intercepts	regression line slope	LOD (μg/mL)	LOQ (μg/mL)
amiloride	*y* = 0.0071× – 0.121	0.9968	0.0065	0.0071	3.02	9.16
chlortiazide	*y* = 0.0204× – 0.015	0.9948	0.0155	0.0204	2.51	7.61
hydrochlortiazide	*y* = 0.028× – 0.1604	0.9959	0.0366	0.028	2.98	9.04
chlorthalidone	*y* = 0.0035× – 0.0045	0.9986	0.0015	0.0035	1.39	4.21
furosemide	*y* = 0.0365× – 0.0117	0.9972	0.0384	0.0365	3.47	10.51

Analysis of the six-point calibration curves (20–220 μg/mL for all diuretics) showed adequate correlation coefficients (*r* > 0.9948), indicating that the method was linear for all five analytes of interest (Table 3). The linearity results were further evaluated using analysis of variance (ANOVA), which confirmed significant linear regression for each diuretic (*F* values: ALM = 4.61 × 10^–17^, CTZ = 5.51 × 10^–19^, HCTZ = 1.32 × 10^–19^, CTD = 4.76 × 10^–18^, and FRS = 7.7 × 10^–21^) with no deviation from linearity.

Method precision was evaluated through repeatability (intraday precision) and interday precision. Intra- and interday precision were evaluated using the coefficient of variation (CV %), and results were analyzed by ANOVA (Table 4). The method demonstrated satisfactory precision at all three concentration levels tested (low, medium, and high), with CV % values of <3.5% for FRS, <4.8% for CTD and HCTZ, <7.9% for AML, and <8.8% for CTZ. These values are well within the guideline limits for complex matrices: up to 20% for low concentrations and up to 15% for medium and high concentrations.

**Table 4:** Precision and Accuracy Results Obtained for Each Diuretic Analyzed

substance of analysis	conc. (μg/mL)	CV %^a^ intraday	CV %* interday	average recovered^b^ (μg/mL)	average recovery (%)
amiloride	60	6.2/2.0/6.2	5.1	57.5 ± 3.2	95.9
	120	5.0/3.4/3.1	3.8	117.2 ± 4.4	97.7
	220	2.9/5.9/1.9	7.9	218.0 ± 5.7	99.1
chlortiazide	60	8.8/5.6/3.3	6.2	60.9 ± 3.5	101.4
	120	2.2/2.2/2.8	4.0	120.6 ± 4.8	100.5
	220	1.0/1.0/0.6	3.6	227.2 ± 4.1	103.3
hydrochlortiazide	60	4.3/4.1/6.6	4.8	59.0 ± 3.2	98.4
	120	3.6/3.6/4.2	3.9	125.1 ± 4.8	104.3
	220	1.6/3.1/1.7	3.4	220.7 ± 5.9	100.3
chlorthalidone	60	4.7/1.9/2.3	3.9	57.7 ± 2.1	96.1
	120	4.5/2.0/3.2	3.1	122.4 ± 3.8	102.0
	220	2.4/1.7/2.7	2.2	226.1 ± 4.9	102.8
furosemide	60	1.4/0.9/2.9	3.3	62.1 ± 2.1	103.4
	120	2.9/2.5/2.0	2.5	124.5 ± 3.1	103.8
	220	0.8/3.0/3.5	2.7	221.8 ± 5.7	100.8

a Coefficient of variation (CV %).

b Mean ± SD.

According to the official guidelines, accuracy should be assessed at a minimum of nine points across three concentration levels (low, medium, and high) within the method’s linear range.^29,30^ It is experimentally determined as the recovery of the reference standard from the spiked matrix. The analytical method demonstrated satisfactory accuracy for all three concentration levels evaluated for the five diuretics, with mean recovery values ranging from 95.9% to 104.3% (Table 4).

The robustness assay was performed by introducing small variations in the eluent flow rate (0.8 and 1.0 mL/min) and oven temperature (33 and 37 °C). Minor changes were observed in the analytes’ retention times (*R*_*t*_) and in the ratio of the analyte peak area to the IS peak area (Table 5). These changes slightly improved chromatographic peak resolution, with all values remaining adequate and above the minimum recommended threshold (>2.0). Variations in the analyte-to-IS peak area ratio resulted in less than 2.0% change in diuretic quantification, confirming the method’s robustness (Table 5).

**Table 5:** Robustness Assay Results Obtained for Each Diuretic Analyzed

substance of analysis	retention time (min)	peak resolution	ratio of areas^a^	concentration (μg/mL)	CV %^b^
amiloride					
*F* = 0.9 mL/min; *T* = 35 °C	7.54	3.5	0.611	100.00	1.5
*F* = 1.0 mL/min; *T* = 35 °C	7.63	3.0	0.592	96.84	
*F* = 0.8 mL/min; *T* = 35 °C	7.41	3.8	0.610	99.78	
*F* = 0.9 mL/min; *T* = 37 °C	7.50	4.2	0.611	99.95	
*F* = 0.9 mL/min; *T* = 33 °C	7.58	3.0	0.612	100.11	
chlorotiazide					
*F* = 0.9 mL/min; *T* = 35 °C^c^	11.21	4.2	2.241	100.04	2.0
*F* = 1.0 mL/min; *T* = 35 °C	11.13	3.5	2.350	104.91	
*F* = 0.8 mL/min; *T* = 35 °C	11.32	4.3	2.234	99.73	
*F* = 0.9 mL/min; *T* = 37 °C	11.15	4.8	2.281	101.83	
*F* = 0.9 mL/min; *T* = 33 °C	11.21	4.1	2.260	100.89	
hydrochlorotiazide					
*F* = 0.9 mL/min; *T* = 35 °C^c^	12.46	5.0	3.251	100.03	2.0
*F* = 1.0 mL/min; *T* = 35 °C	12.32	4.5	3.330	102.46	
*F* = 0.8 mL/min; *T* = 35 °C	12.51	5.5	3.156	97.11	
*F* = 0.9 mL/min; *T* = 37 °C	12.30	5.8	3.230	99.38	
*F* = 0.9 mL/min; *T* = 33 °C	12.52	4.9	3.280	100.92	
chlorothalidone					
*F* = 0.9 mL/min; *T* = 35 °C^c^	19.00	5.6	0.371	100.27	2.0
*F* = 1.0 mL/min; *T* = 35 °C	18.86	5.4	0.361	97.57	
*F* = 0.8 mL/min; *T* = 35 °C	19.08	5.9	0.351	94.86	
*F* = 0.9 mL/min; *T* = 37 °C	19.00	5.9	0.356	96.22	
*F* = 0.9 mL/min; *T* = 33 °C	18.93	4.8	0.360	97.30	
furosemide					
*F* = 0.9 mL/min; *T* = 35 °C^c^	19.94	7.7	3.781	100.03	1.5
*F* = 1.0 mL/min; *T* = 35 °C	19.84	7.2	3.752	99.26	
*F* = 0.8 mL/min; *T* = 35 °C	20.05	7.8	3.805	100.66	
*F* = 0.9 mL/min; *T* = 37 °C	19.98	7.9	3.671	97.12	
*F* = 0.9 mL/min; *T* = 33 °C	19.80	7.5	3.703	97.96	

a Ratio of the analyte peak area to the internal standard (IS) area.

b Coefficient of variation (CV %).

c Original method: flow 0.9 mL/min, temperature 35C°.

The matrix effect is defined as the interference in the analysis of target substances caused by matrix components. It can be assessed by comparing the analytical response of the complex matrix spiked with the reference standard to that of the reference standard alone. When the difference in response is within ±20%, the matrix effect is considered not significant.^34,35^ Regression lines for all diuretics, analyzed either alone or in the presence of the matrix, showed linear responses (*r* > 0.99) (Table 6 and Figure 3). Figure 3 illustrates the parallelism of the regression lines from the matrix effect test, indicating no interference from matrix components. Furthermore, statistical evaluation of the variance of the *y*-intercept (b) and slope (a) of the regression lines, using a *t*-test, revealed no significant differences (*p* > 0.05 for both a and b) (Table 6).

**Table 6:** Matrix Effect was Assessed by Comparing the Linear Regression Analyses of the Reference Standards with Those of the Matrix Spiked with Reference Standards

substance	mean regression line (matrix extract)	*r* (matrix)	mean regression line (reference standard)	*r* (reference standard)	*p* ^a^ (a)	*p* ^a^ (b)
amiloride	*y* = 0.0071× – 0.121	0.9968	*y* = 0.0075× – 0.0719	0.9989	0.0929	0.3494
chlorotiazide	*y* = 0.0204× – 0.015	0.9948	*y* = 0.0211× – 0.0021	0.9957	0.1175	0.2590
hydrochlorotiazide	*y* = 0.028× – 0.1604	0.9959	*y* = 0.0286× – 0.0969	0.9990	0.1355	0.8459
chlorthalidone	*y* = 0.0035× – 0.0045	0.9986	*y* = 0.0036× + 0.0089	0.9989	0.1347	0.7791
furosemide	*y* = 0.0365× – 0.0117	0.9972	*y* = 0.0367× + 0.097	0.9988	0.2302	0.4168

a *p* > 0.05, no significant difference for the comparison of the angular (a) and linear (b) coefficients of the mean regression line equations obtained for reference standards and the supplemented matrix.

**Figure 3:**
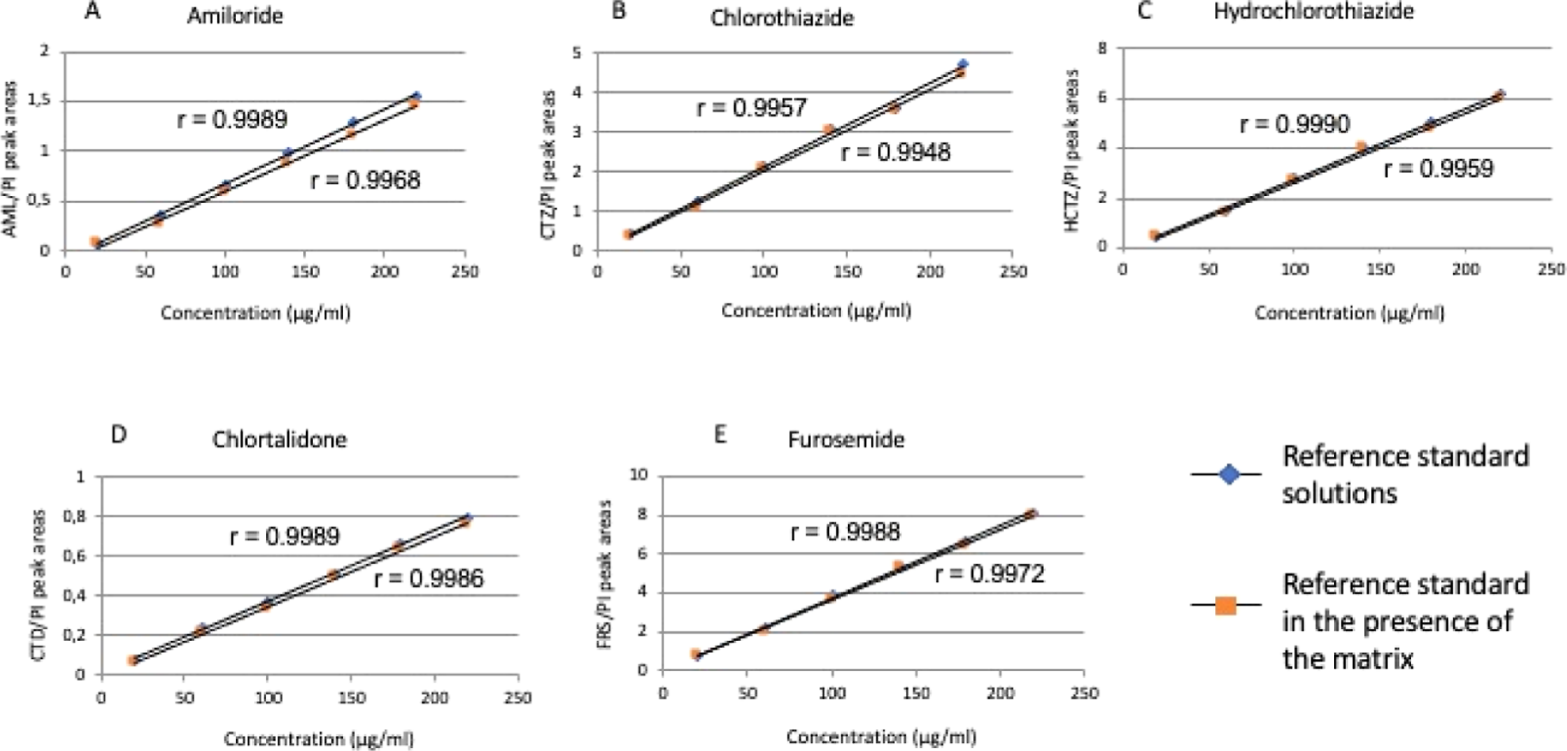
Calibration curves for each diuretic were constructed in the complex matrix spiked with reference standard solutions (orange dots) and in the reference standards alone (blue dots) to evaluate the matrix effect. The *X*-axis represents analyte concentration (20–220 μg/mL), and the *Y*-axis represents the ratio of the mean areas of the diuretics to the IS. Regression lines for all diuretics (A–E), analyzed either alone or in the presence of the matrix, showed linear responses (*r* > 0.99) and similar profiles, indicating no interference from matrix components. Statistical evaluation of the variance of the *y*-intercept and slope revealed no significant differences (*t*-test, *p* > 0.05).

### 3.2 Stability of Extracted Samples

Stability is defined as the period during which a sample or product maintains the same characteristics it had at the time of manufacture, within specified limits. The stability of chemical products depends on environmental factors, such as temperature, humidity, and light, as well as product-related factors, including the physical and chemical properties of the active substances and other components of the sample.^31^

The stability of the matrix extract was evaluated at three concentration levels (60, 120, and 220 μg/mL) to determine whether the analytes’ concentrations changed during analysis. All diuretics remained stable after 30 h in the autosampler, 48 h on the benchtop, or up to 7 days in the refrigerator, as indicated by recovery values above 94% and no significant differences compared to freshly prepared samples (control, *p* > 0.05) (Table 7).

**Table 7:** Results of the Stability Test Performed with the Analyzed Product Immediately after the Extraction Process from the Matrix (Control), and after Exposure to the Different Storage Conditions

substance (μg/mL)	% recovered from control	% recovered from refrigerator^a^	*p*	% recovered from benchtop^b^	*p*	% recovered from autosampler^c^	*p*
amiloride							
60	99.46	100.03	0.243	100.20	0.375	100.02	0.218
120	96.67	95.70		97.08		96.07	
220	99.44	101.03		101.0		102.03	
chlortiazide							
60	100.59	98.51	0.268	98.54	0.256	96.81	0.247
120	99.08	97.81		101.02		97.78	
220	100.50	98.81		98.51		99.71	
hydroclortiazide							
60	98.53	95.39	0.135	100.08	0.355	99.81	0.106
120	97.58	98.96		98.56		95.03	
220	96.61	100.01		97.51		94.95	
chlorthalidone							
60	94.81	95.30	0.300	97.52	0.168	94.02	0.499
120	101.02	98.40		99.02		100.3	
220	95.86	99.50		96.03		99.08	
furosemide							
60	105.02	100.1	0.195	99.05	0.189	101.02	0.429
120	102.03	101.30		98.72		99.75	
220	99.98	98.80		96.78		96.74	

a Refrigeration time: 4–8 °C for 7 days.

b Benchtop (25 °C) for 48 h.

c Equipment autosampler (35 °C) for 30 h. *p* > 0.05, no significant difference.

### 3.3 Analysis of Commercial Samples by CLAE-UV

The validated analytical method was used to analyze 21 commercial WPDS samples from consumers in Porto Alegre, RS, Brazil. Among the samples, 57.1% (12) tested negative for all diuretics, while 42.9% (9) tested positive for at least one diuretic. Of the positive samples, 28.6% (6) were positive for one diuretic, 9.5% (2) for two diuretics, and 4.8% (1) for three diuretics (Figure 4).

**Figure 4:**
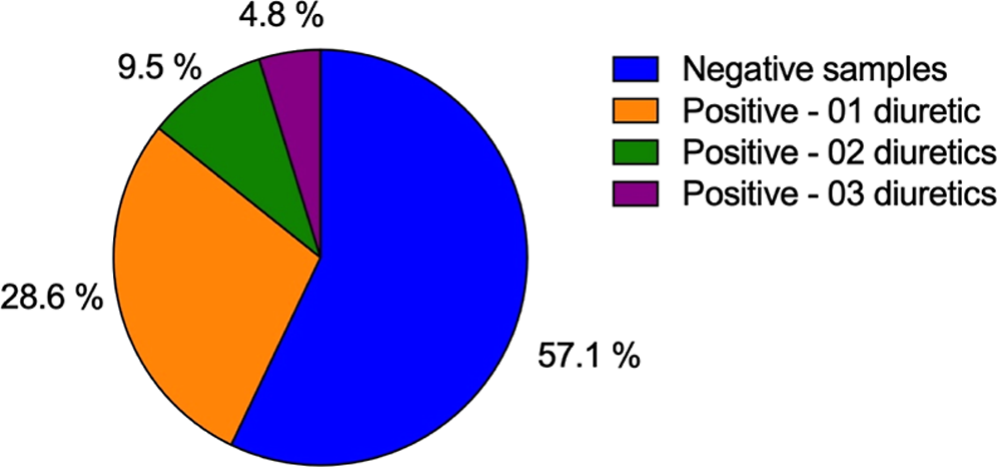
Results of analyses performed on commercial WPDS samples from consumers in the metropolitan region of Porto Alegre, RS, Brazil.

Among the positive tested samples, the most frequently detected diuretic was FRS, found in 5 samples (55.6%), followed by HCTZ (4 samples, 44.4%) and CTZ (3 samples, 33.3%). AML was detected in only 1 sample (11.1%), while CTD was not detected in any of the analyzed samples. In sample AM19, three diuretics—FRS, HCTZ, and CTZ—were detected (Figure 2B).

According to the literature, it is common to detect contaminants in dietary supplements, including diuretics.^5,16–21,36,37^ FRS was the most frequently detected diuretic in the analyzed commercial samples, likely due to its higher diuretic potency among the compounds investigated. In addition, it is rapidly excreted from the body, making it difficult to detect in antidoping tests.^22^ The second most frequently detected diuretic was HCTZ, which is widely used in Brazil due to its low cost and easy availability.^38^ Corroborating our data, Müller et al. reported the presence of hydrochlorothiazide (17.65–192.91 mg/day consumption) and furosemide (91.5 mg/day consumption) as adulterants in dietary supplements.^18^ Kozhuharov et al. also reported the presence of furosemide (2.42 mg/day consumption),^20^ while Lee et al. detected hydrochlorothiazide and furosemide in commercial dietary supplements at concentrations ranging from 0.051–162 mg/g.^17^

Another diuretic detected in the WPDS samples was CTZ, which is not registered with ANVISA and cannot be marketed for human use in Brazil.^39^ However, this active ingredient is registered and available in Canada and the United States.^22^ Its presence in the samples may be related to the fact that most WPDS are not produced in Brazil and are often purchased online from other countries.^40^

The concentrations of diuretics detected in the positive samples varied considerably among the WPDS products, as shown in Table 8. The manufacturer’s recommended daily dose of WPDS is approximately 35 g, taken pre- or postexercise. Based on this dosage, the amount of diuretics consumed by WPDS users was either subtherapeutic or within the recommended therapeutic range.

**Table 8:** Results of Analyses of Positive WPDS Samples for the Diuretics of Interest. The Results are Expressed as mg of Analyte/g WPDS and Correlated to the Manufacturer’s Recommended Daily Dose of WPDS

sample	analyte content (mg/g)	diuretic content relative to the recommended daily dose of WPDS (mg/35 g)
furosemide		
AM9	0.20	7.0
AM13	1.82	63.7
AM14	0.52	18.2
AM19	0.62	21.7
AM20	0.28	9.8
hydroclortiazide		
AM4	1.40	49.0
AM6	0.66	23.1
AM13	0.33	11.6
AM19	0.25	8.8
chlortiazide		
AM6	1.87	64.8
AM15	0.22	7.7
AM19	0.23	8.1
amiloride		
AM16	0.23	8.1

The highest FRS content detected was 63.7 mg per 35 g of WPDS, whereas the recommended therapeutic oral dosage for adults varies significantly by indication, ranging from 20 to 240 mg/day, with a maximum daily dose of 600 mg. For HCTZ, the highest amount detected was 49.0 mg/35 g of WPDS, compared with a therapeutic dosage of 12.5–100 mg/day and a maximum of 200 mg/day. For CTZ, the highest amount detected was 64.8 mg/35 g of WPDS, while the recommended therapeutic range is 250–1000 mg/day. AML was detected at 8.1 mg/35 g of WPDS, with a recommended therapeutic dosage of 5–10 mg/day.^41^ Although most results reflect doses below the typical therapeutic range, these levels are sufficient to elicit diuretic effects and can be detected in antidoping tests, given the increasing sensitivity of the current analytical techniques.^9^

Some samples contained more than one diuretic, consistent with literature reports of contamination in dietary supplements and the associated risks to consumer health.^8,17,18,42,43^ The side effects of diuretics include loss of appetite, itching, blurred vision, headache, stomach upset, weakness, and dizziness. Furthermore, diuretics may cause changes in the electrolyte levels (potassium and others), dehydration, renal stress, cardiovascular strain, especially when combined with intense exercise or in individuals with underlying health conditions.^24^ As mentioned earlier, Figure 2B shows the chromatographic profile of a sample in which three different diuretics were detected, potentially enhancing the drugs’ side effects and increasing health risks.

Currently, legislation does not require the analysis of diuretic compounds in WPDS. Nevertheless, ensuring that these products are free from substances banned in sports is essential to protect athletes’ health and integrity. The use of dietary supplements among athletes continues to rise, and as mentioned earlier, several cases have reported positive antidoping results following the consumption of contaminated products.^16,17,20–22^ Therefore, monitoring and quantifying such compounds are critical to prevent athletes’ inadvertent exposure to banned substances.

## 4 Conclusions

In this study, an analytical method using HPLC-UV was successfully developed and validated for the simultaneous determination of five diuretic adulterants in WPDS: AML, CTZ, HCTZ, CTD, and FRS. The method proved to be selective, linear, accurate, precise, and robust for the identification and quantification of undeclared diuretics commonly used as WPDS adulterants. Additionally, the method is simple, fast, cost-effective, and can be easily implemented in routine quality control.

The high contamination rate of 42.9% for at least one analyzed diuretic highlights the risk athletes face when using dietary supplements. Accordingly, this study underscores the importance of proper quality control to prevent unintentional doping and reduce associated health risks. The safety issue regarding dietary supplements is authentic, and an improvement of the current legislation regulating dietary supplements compatible between countries is needed to ensure the safety, efficacy, and legality of the available nutritional supplements.

## Supplementary Material



## Notes

Chemical structure and ultraviolet (UV) spectrum of each analyzed diuretic, experimental determination of the limit of quantification (LOQ) (PDF)
