# Supplementary material for: Detection of Diuretics Contamination in Whey Protein-Based Dietary Supplements
Source: ACS Omega. 2026 Feb 5;11(6):9388–97. doi: 10.1021/acsomega.5c09357 (PMC12917619; doi:10.1021/acsomega.5c09357)
Supplement: Supplementary file 1 [file ao5c09357_si_001.pdf]

DETECTION OF DIURETICS CONTAMINATION IN WHEY PROTEIN-BASED DIETARY SUPPLEMENTS

Inélia Maria Franskoviaki[1], Pâmela Cristina Lukasewicz Ferreira[1], Vanessa Klimkowski Argoud[1], Pedro Eduardo Froelich[1], Aline Rigon Zimmer[1*].

Supplemental material

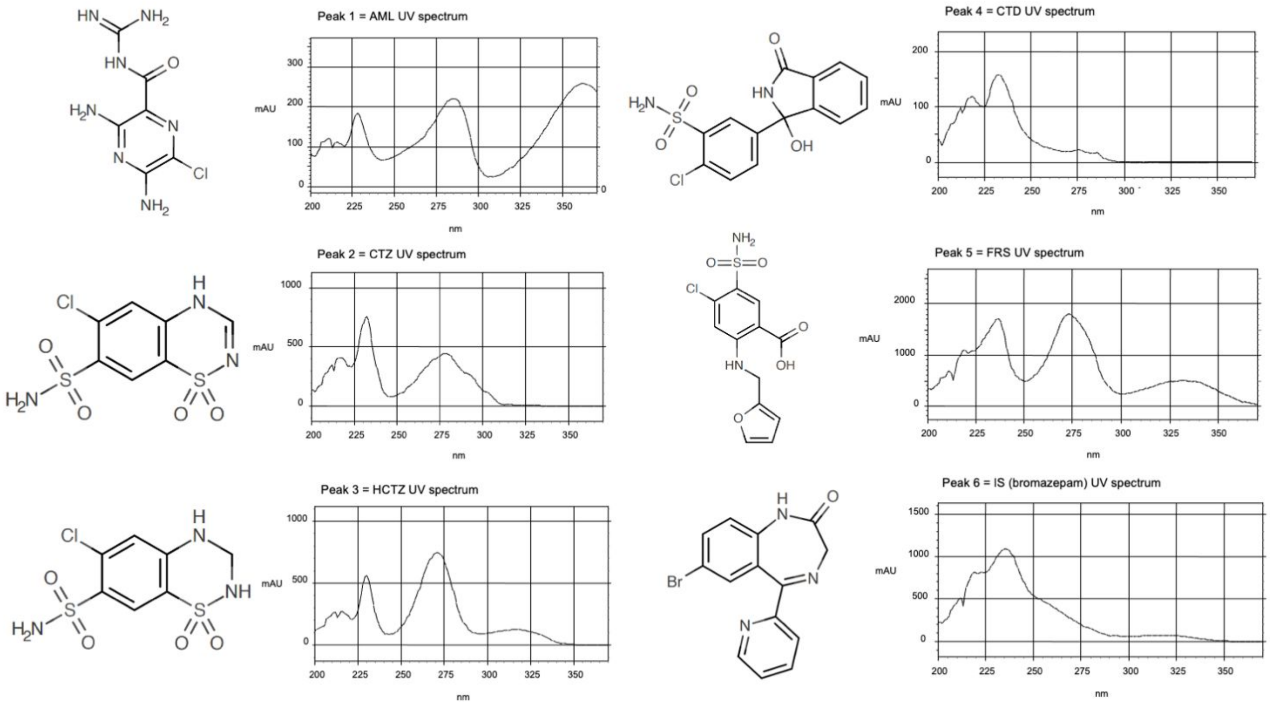

Figure S1 - Chemical structure and UV spectrum of each analyzed diuretic obtained by DAD detector scanning at the chromatographic peak. Peak 1) AML = Amiloride, Peak 2) CTZ = Chlorothiazide, Peak 3) HCTZ = Hydrochlorothiazide, Peak 4) CTD = Chlorthalidone, Peak 5) FRS = Furosemide, Peak 6) IS = Bromazepam. Chemical structures were drawn with Marvin Sketch 25.3.2, Chemaxon.

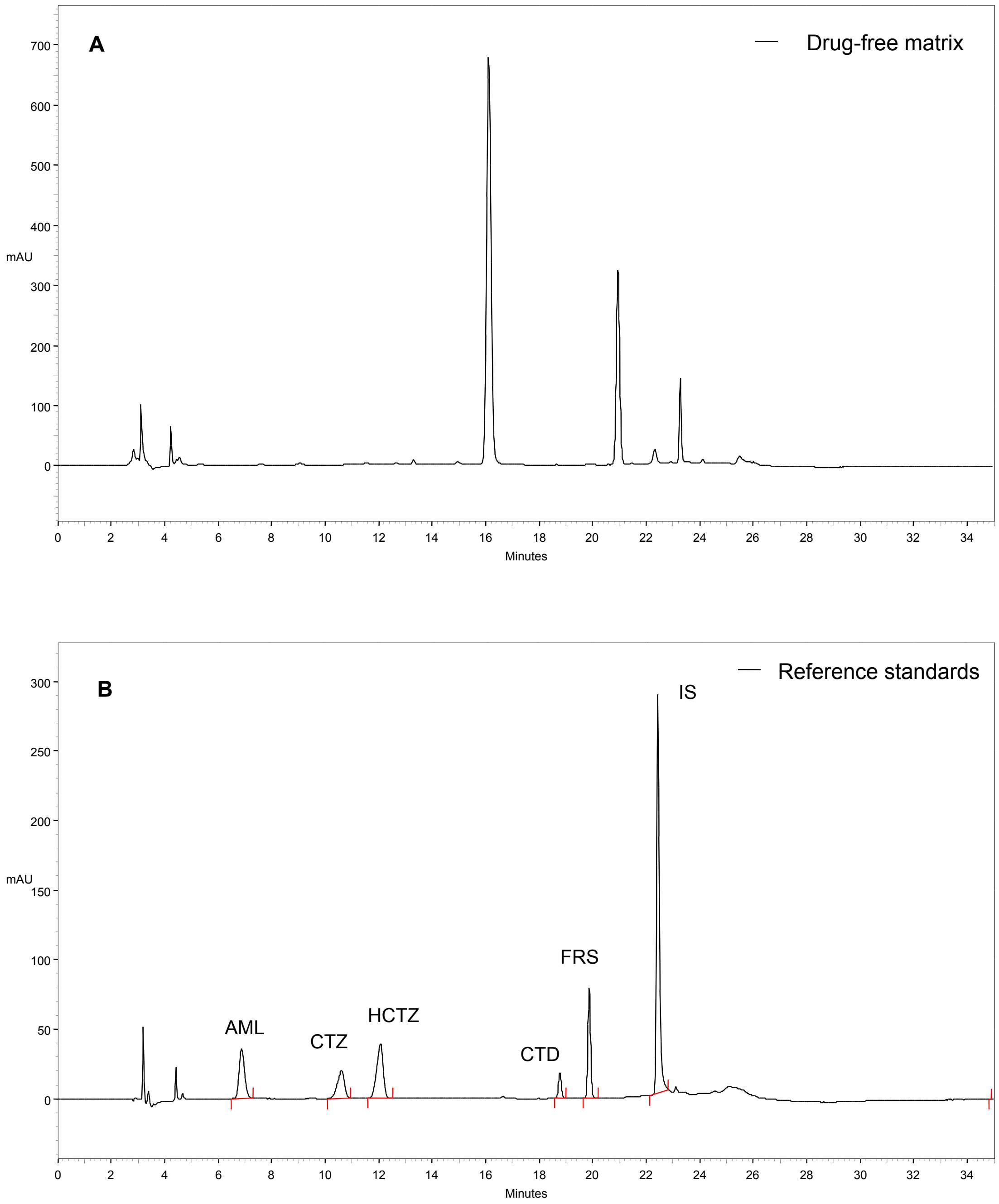
A
Drug-free matrix
mAU
Minutes
B
Reference standards
AML
CTZ
HCTZ
CTD
FRS
IS
mAU
Minutes

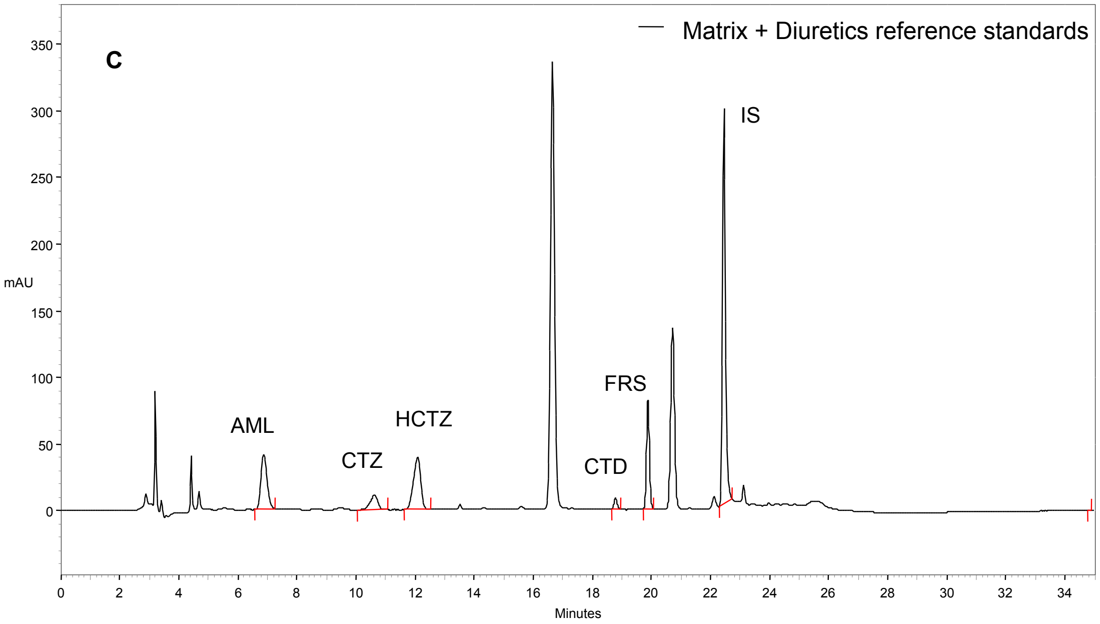

D

Substance of analysis	LOQ (µg/ml) estimated	LOQ (µg/ml) experimental (mean + SD)	Precision (CV%)	Recovery (%)
**Amiloride**	9.16	9.27 ± 0.11	1.2	101.2
**Chlorothiazide**	7.61	7.22 ± 0.09	1.2	94.9
**Hydrochlorothiazide**	9.04	9.14 ± 0.21	2.3	101.1
**Chlorthalidone**	4.21	4.36 ± 0.17	3.9	103.5
**Furosemide**	10.51	10.77 ± 0.36	3.4	102.5

Figure S2 – Experimental determination of the limit of quantification (LOQ). **A)** Chromatographic profile of the drug-free matrix. **B)** Chromatographic profile of the diuretic reference standards at the LOQ concentrations: AML = Amiloride, 9.16 µg/mL, Retention time (Rt) = 6.87 min; CTZ = Chlorothiazide, 7.61 µg/mL, Rt = 10.62 min; HCTZ = Hydrochlorothiazide, 9.04 µg/mL, Rt = 12.09 min; CTD = Chlorthalidone, 4.21 µg/mL, Rt = 18.77 min; FRS = Furosemide, 10.51 µg/mL, Rt = 19.87 min; IS = Bromazepam, 100 µg/mL, Rt = 22.44 min. **C)** Chromatographic profile of the extraction product from drug-

free matrix supplemented with reference standards of the diuretics AML (Rt = 6.87 min), CTZ (Rt = 10.65 min), HCTZ (Rt = 12.10 min), CTD (Rt = 18.78 min), FRS (Rt = 19.88 min) at the LOQ concentration, and IS (Rt = 22.45 min), showing no overlap between matrix peaks and the diuretics analyzed at the LOQ. **D)** Comparison of the estimated and experimentally determined LOQ values (n = 3), demonstrating suitable precision (CV = 1.2–3.9%) and accuracy (94.9–103.5%).
